# Integration Method of Microchannel and Vertical Micromesh Structure for Three-Dimensional Cell Culture Using Inclined Exposure and Inclined Oxygen Ashing

**DOI:** 10.3390/mi9120681

**Published:** 2018-12-19

**Authors:** Hidetaka Ueno, Kou Yamada, Takaaki Suzuki

**Affiliations:** 1 Division of Mechanical Science and Technology, Gunma University, Kiryu 376-8515, Japan; t162b001@gunma-u.ac.jp (H.U.); yamada@gunma-u.ac.jp (K.Y.); 2 JST, PRESTO, Kawaguchi 332-0012, Japan

**Keywords:** vertical porous membrane, inclined exposure, inclined oxygen ashing, assembly-free, SU-8, biomicrofluidics

## Abstract

Culturing cellular tissues inside a microchannel using an artificial three-dimensional (3D) microstructure is normally conducted to elucidate and reproduce a biological function. The thick photoresist SU-8, which has a microscale resolution and high aspect ratio, is widely used for the fabrication of microchannels and scaffolds having 3D structures for cell culture. However, it is difficult to accurately fabricate a mesh structure with a pore size that is smaller than the cells that has an overall height greater than 50 μm because of the deterioration of the verticality of exposure light and the diffusion of acid, which accelerates the crosslinking reaction in the SU-8 layer. In this study, we propose a method of integrating a vertical porous membrane into a microchannel. The resolution of the vertical porous membrane becomes more accurate through inclined oxygen ashing, without degrading the robustness. Because a single mask pattern is required for the proposed method, assembly error is not generated using the assembly-free process. The fabricated vertical porous membrane in the microchannel contained micropores that were smaller than the cells and sufficiently robust for a microfluidic system. HepG2 cells were attached three-dimensionally on the fabricated vertical porous membrane to demonstrate 3D cell culture.

## 1. Introduction

The purpose of cell culture inside a microchannel or a scaffold of microstructures is to elucidate and reproduce biological functions by creating biological tissues inside an artificial environment. Inside the human or animal body, multiple types of cells are arranged three-dimensionally. By mimicking the three-dimensional (3D) cellular tissue in a microchannel, not only is the biological function elucidated, but a medical and chemical assay can also be conducted without animal tests [1,2,3,4,5,6]. Because cell culture inside a microchannel fabricated by microfabrication method for fabricating semiconductor can minimize the amount of culture medium and control the flow rate and pressure easily, this technique is suitable for fabricating high-density and stable biological models. Meanwhile, because cell culture on scaffolds such as thin membranes or porous structures has the advantage of enabling control of the shape and size of cellular tissues by designing the scaffold, it is easy to construct two-dimensional (2D) and 3D cellular tissues. In addition, because cellular tissues with a complex structure are constructed by simple cell seeding on the scaffold, it can reduce the risk of contamination. 

Researchers have used the advantages of cell cultures both inside a microchannel and on a scaffold. Chung et al. [7] and Osaki et al. [8] created cellular tissues on microstructures integrated or fabricated inside a microchannel. Meanwhile, Esch et al. [9] and Satoh et al. [10] assembled cellular tissue pre-cultured on a scaffold into a microchannel. However, when integrating microstructures inside a microchannel or insert scaffold, it is difficult to fabricate 3D cellular tissues because of problems such as difficult assembly, low resolution, or low aspect ratio. When a scaffold with pre-cultured tissues is assembled into a microchannel, it is difficult to create high-density cellular tissues because of the dead volume. When 3D structures are accumulated in the microchannel in advance by 2D semiconductor manufacturing technology, it is difficult to achieve a resolution lower than the cell size and a height necessary for cell loading and 3D culture. 

A thick photoresist, SU-8, having micrometer-scale resolution and submillimeter-scale coated thickness is often used for fabricating 3D microstructures [11,12,13,14]. Moreover, because SU-8 exhibits chemical stability and cell culturing capability as a scaffold material for cells [15], it is possible to create complex artificial cellular tissues by seeding cells on the SU-8 structure [16,17,18]. However, because the attenuation of exposure light and the diffusion of acid accelerate the crosslinking reaction in the SU-8 film thickness direction, the resolution of an SU-8 structure decreases with increasing thickness. Therefore, it is difficult to fabricate a vertical porous membrane with a height of over 50 μm and a pore size smaller than the cell size. 

In this research, we propose an assembly-free integration method for fabricating a high-resolution vertical porous membrane using inclined exposure and inclined oxygen ashing. The closed micropores in the membrane with a decreasing resolution of a thick film of SU-8 were treated uniformly using inclined oxygen ashing, and high-resolution pores on the vertical porous membrane were obtained without fabrication errors such as sticking. Moreover, because the vertical porous membranes and microchannels were fabricated simultaneously, fabrication error during assembly did not need to be considered. To confirm the usability of the proposed method for fabricating the microchannel for 3D cell culture, a cell culturing test was performed on the fabricated vertical porous membrane.

## 2. Method

### 2.1. Principle of Vertical Porous Membrane Integration Method Using an Inclined Exposure Method and Inclined Oxygen Ashing

**Figure 1:**
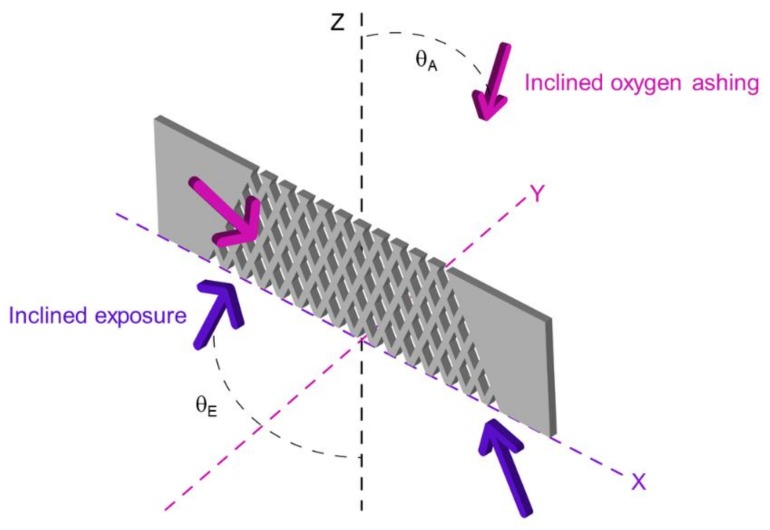
Principle of inclined exposure and inclined oxygen ashing. The inclined exposure is applied from the back side of the substrate, which contains a pattern. The mesh structure on the vertical porous membrane is fabricated by inclined exposure, the angle of which is θ_E_. The angle of inclined ashing is from a 90° to the *Z* axis. The inclination angle θ_A_ is determined such that etching is performed on the etched vertical porous membrane.

In this paper, we propose an integration method for fabricating a vertical porous membrane inside a microchannel using inclined exposure and inclined oxygen ashing. The principle of the proposed integration method is shown in Figure 1. In this method, SU-8 photoresist on a glass substrate is partially exposed through patterned metal on the glass substrate by simple inclined exposure to fabricate a vertical porous membrane and microchannel walls along the pattern and surface of the developed SU-8, which is treated by inclined oxygen ashing to open the micropores of the porous membranes. Because the inclined exposure uses a mask pattern on a glass substrate, the contact gap generated by edge beads of the thick photoresist coated on the substrate need not be considered. Inclined oxygen ashing is conducted to improve the ratio of open micropores on the vertical porous membranes. Because oxygen plasma has a high oxidizability, it can etch the surface of the vertical porous membrane and make it porous. In conventional methods, because inclined plasma is used for selective etching, it is necessary to fabricate micropatterns in advance. As the proposed method is used for etching the surface of a vertical porous membrane, the patterning process is not required [19]. In this research, in order to etch the side of the vertical porous membrane, an angle is created between the etching stage and the substrate. By creating the angle, the oxygen plasma is irradiated on the side surface of the vertical porous membrane, even though it is placed in parallel. Using the proposed method, a high-resolution vertical porous membrane can be fabricated easily.

### 2.2. Inclined Backside Exposure

The minimum size fabricated by the inclined exposure was evaluated using the dot pattern size, interval, and exposure dose as the parameters. The machining accuracy primarily depends on the attenuation and the scatter of exposure light in the thickness direction of SU-8. Because the exposure light was scattered at the height of several tens of micrometers from the surface of the mask, the resolution of the structure decreased as the height of the fabrication structure increased. Therefore, the optimum value of the resolution and the height of structure—which is a tradeoff relationship—were evaluated through an exposure test.

On the sample for exposure evaluation, the optimal exposure dose differed according to the change in the pattern size. Therefore, a dot pattern as a lattice structure on the porous membrane was used as the sample pattern. The dot size, which decides the size of the lattice, was from 1 to 6 μm, and the interval of the dot patterns was from 5 to 26 μm. In the backside exposure process, the exposure light passed through air, glass, and SU-8, thereby exhibiting different refraction indices. Considering the change in the angle of the exposure light, the incident angle of θ_E_ was set to 30°. The vertical porous membrane was fabricated in two exposure steps, with the symmetrical axis perpendicular to the substrate. The total exposure dose of the two exposure steps in the evaluation experiment was set from 300 to 600 mJ/cm^2^ at intervals of 100 mJ/cm^2^. 

**Figure 2:**
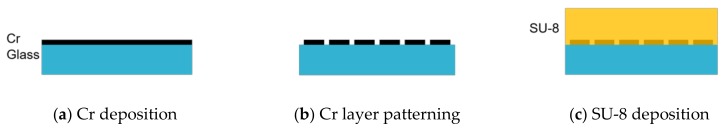

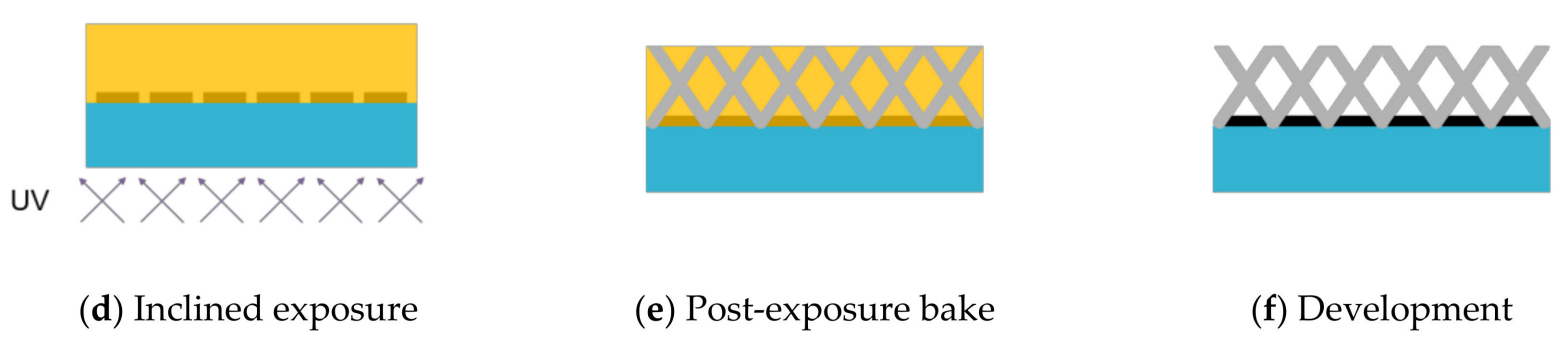
Process flow for vertical porous membrane on Cr patterned glass substrate by inclined exposure. The vertical porous membrane was fabricated by inclined exposure from the back side of the substrate. Because patterns created in advance using Cr patterns were used, a contact gap was not generated between the Cr pattern and SU-8, and no alignment was necessary.

The fabrication process of the sample for evaluating the size of the vertical porous membrane is shown in Figure 2. First, a Cr layer was deposited on a glass substrate. Dot patterns were fabricated on the Cr layer by photolithography and wet etching. An SU-8 3005 (MicroChem Corp., Westborough, MA, USA.) layer was deposited by a spray coater. After removing the solvent through soft baking, the SU-8 layer was exposed twice from the back side of the substrate. After a post-exposure bake at 95 °C for 10 min, the SU-8 layer was developed. The fabricated structure was observed by scanning electron microscope (SEM, JCM-5700LV, JEOL Ltd., Tokyo, Japan).

### 2.3. Double-Sided Inclined Oxygen Ashing

The ability of inclined oxygen ashing to increase the ratio of open micropores on the vertical porous membrane was evaluated. It is difficult to balance between resolution and robustness by changing the dot size and exposure dose. Therefore, double-sided inclined oxygen ashing was adopted to obtain the vertical porous membrane with open micropores.

**Figure 3:**
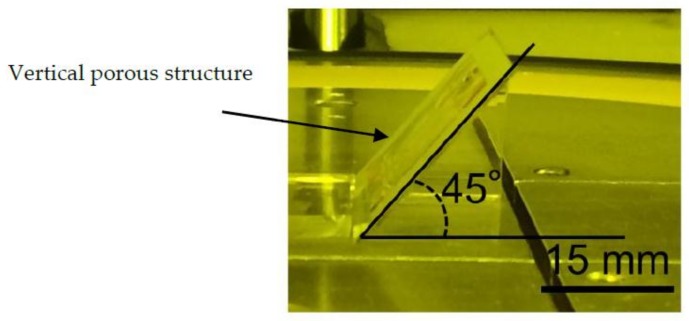
Inclined oxygen ashing method. Oxygen plasma irradiates the vertical porous membrane, inclined at 45° on an ashing stage. Ashing was performed twice from both sides of the vertical porous membrane.

In oxygen ashing, activated oxygen moves in the direction of the ions. Therefore, the etched surface should be placed in the ion direction. For etching multiple layers simultaneously, the vertical porous membrane was etched at a 45° angle, as shown in Figure 3. The samples for evaluation were fabricated using the dot pattern size:interval settings 3:5, 3:7, and 3:9 μm. Since the dot pattern is square, the dot pattern size refers to the size of one side of the square. The interval means the distance between adjacent squares. The fabrication process was the same as that of the sample for exposure evaluation, with the exposure dose of 600 mJ/cm^2^. The fabricated sample was inclined and etched by oxygen plasma at the RF power of 200 W, temperature of 60 °C, oxygen flow rate of 110 sccm, and pressure of 40 Pa. After ashing, the fabricated structure was observed using SEM.

## 3. Results

### 3.1. Fabrication of Vertical Porous Membrane Using Inclined Exposure

**Figure 4:**
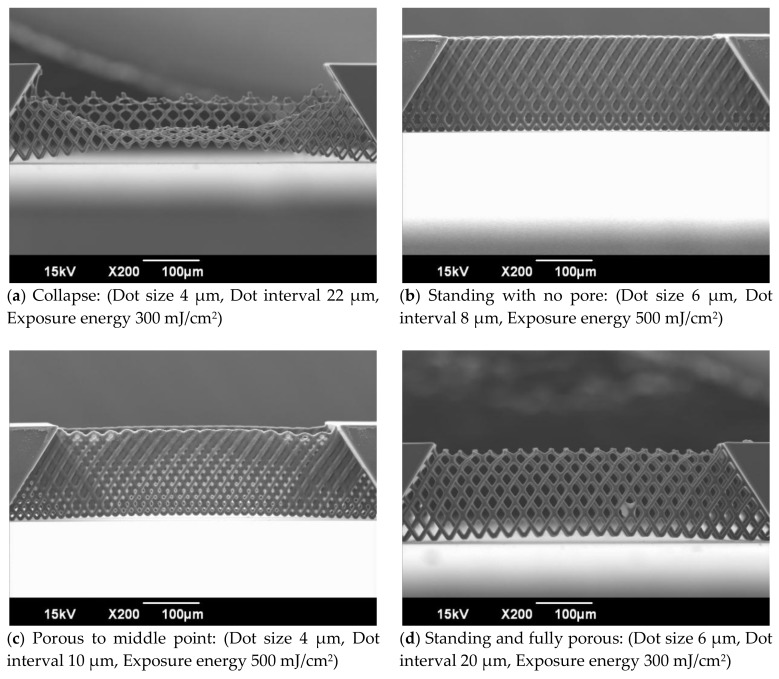
SEM images of the vertical porous membrane fabricated using inclined exposure. The vertical porous membrane was fabricated by changing the dot size, interval, and exposure dose. The fabricated structures are divided to (**a**) collapse, (**b**) standing with no pore, (**c**) porous to middle point, or (**d**) standing and fully porous.

**Figure 5:**
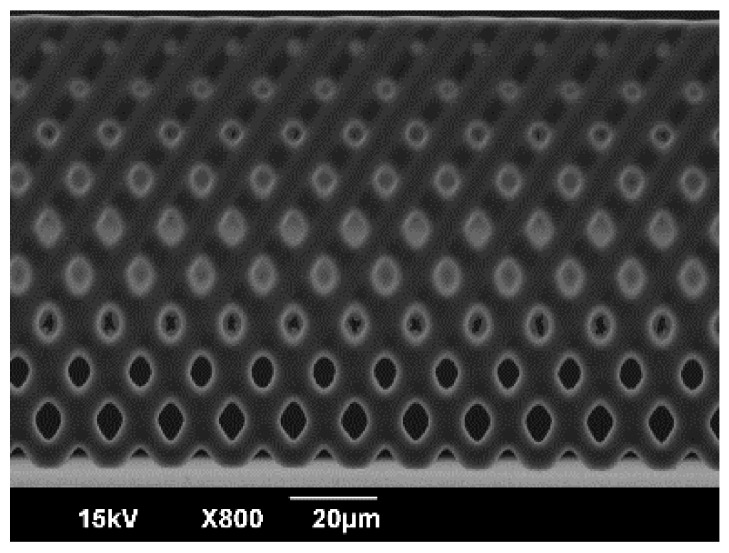
SEM image of the enlarged view of a membrane which is porous to middle point. The pore in the case of “porous to middle point” is closed by a thin film.

SEM images of the vertical porous membrane fabricated using inclined exposure without inclined oxygen ashing are shown in Figure 4. The resolution and robustness of the vertical porous membrane depends on the dot size, interval, and exposure dose. While decreasing the exposure dose, the resolution became higher and the robustness became lower. Additionally, the structure collapsed when the dot size was small, the interval was long, or the exposure dose was low (Figure 4a). Meanwhile, the pore on the vertical porous membrane did not open with a high exposure dose (Figure 4b). The exposure intensity attenuated as the distance from the substrate increased. Therefore, the size of the pores became smaller and closed gradually, and the fabricated structure was porous until the middle point (Figure 4c). When an exposure dose that was suitable for the dot size and interval was used, the fabricated vertical porous membrane contained pores and was robust (Figure 4d). An enlarged view of the porous membrane to the middle point is shown in Figure 5. As shown, the closed pore was closed by the thin film.

**Figure 6:**
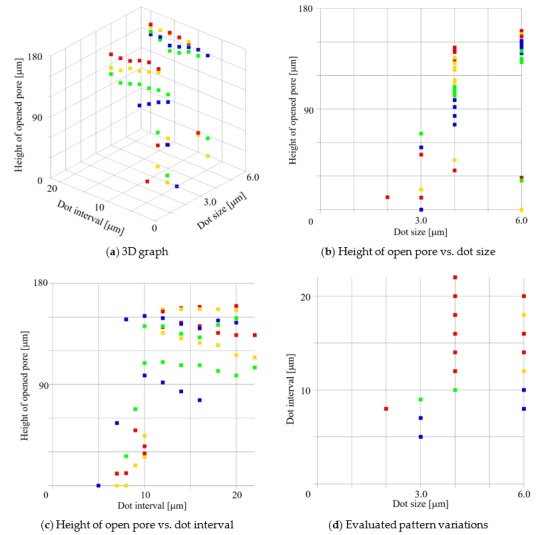
Height of the open pores from the substrate as a function of the dot size, interval, and exposure dose. The *X* axis is the dot size, *Y* axis is the dot interval, and *Z* axis is the distance from the substrate to the open pore. The color of each point indicates the exposure dose. The blue, green, yellow, and red points are 300, 400, 500, 600 mJ/cm^2^, respectively.

Figure 6 shows the height of the opened pore from the surface of the substrate as a function of the dot size, interval, and exposure dose. The *X* axis is the dot size, the *Y* axis is the dot interval, the *Z* axis is the distance from the substrate to the opened pore. The color of each point indicates the exposure dose. The blue, green, yellow, and red points correspond to 300, 400, 500, and 600 mJ/cm^2^, respectively. The collapsed or vertical porous membrane had no opened pore such as the structure shown in Figure 4a,b and hence is not shown in the graph. It is clear that a higher exposure energy is required for creating a higher vertical porous membrane. Meanwhile, too much exposure energy rendered closed pores.

### 3.2. Evaluation of the Inclined Oxygen Ashing after Inclined Exposure

**Figure 7:**
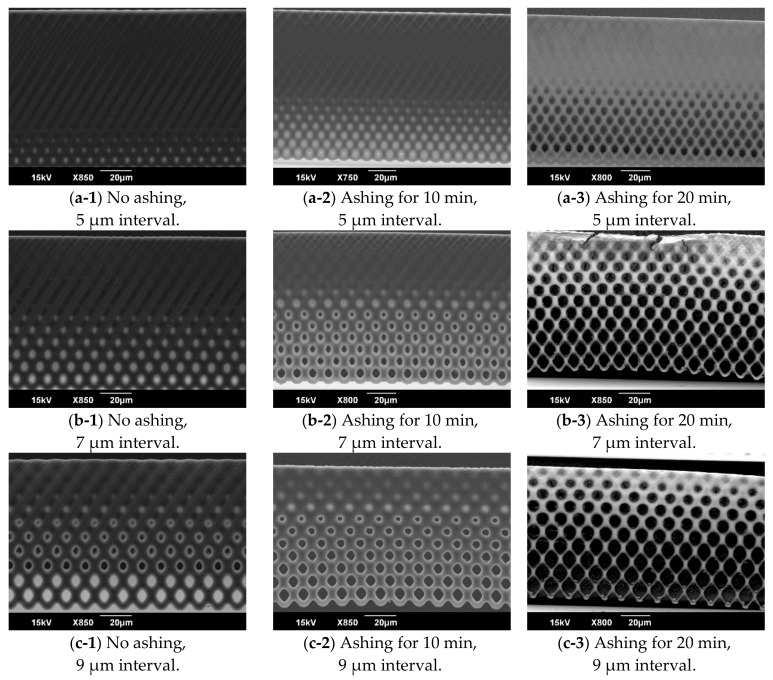
SEM images of the vertical porous membrane inclined oxygen ashing. To evaluate the effect of the inclined oxygen ashing, a fabricated vertical porous membrane with a dot size of 3 μm was etched.

For evaluating the effect of the inclined oxygen ashing, a sample fabricated by an inclined exposure with the exposure dose of 600 mJ/cm^2^ was prepared. The dot size was 3 μm, and the interval was from 5 to 9 μm. The inclined ashing time was 10 or 20 min on a single side. The SEM images of the vertical porous membrane etched by inclined oxygen ashing are shown in Figure 7. Particularly, for the pores closed by the thin film on the vertical porous membrane, the dot size of 7 or 9 μm was opened by the inclined oxygen ashing. Meanwhile, the pores on the vertical porous membrane fabricated using the dot pattern of 5 μm were not opened, even after the inclined oxygen ashing. Through inclined oxygen ashing for 20 min, not only the closed pores but also the vertical porous membrane were etched and collapsed.

## 4. Application for Biomicrofluidics

### 4.1. Method for Integration of Vertical Porous Membrane in Microchannel

Microchannels allow easy control of the internal environment, and are used for multiple kinds of biological experiments. Meanwhile, porous membranes contain numerous micropores and are used for scaffolds to seed cells. Because a porous membrane can pass through a material metabolized by cells, it can mimic a barrier tissue [20,21,22]. However, when porous membranes are arranged in a microchannel with microscale intervals, it is difficult to set the porous membranes with the intended interval, owing to the problem of alignment accuracy.

Inclined exposure is useful not only for fabricating vertical porous membranes, but also for the fabrication of microchannels [23]. Moreover, by creating the micropattern for fabricating vertical porous membranes and microchannels on the same layer, a vertical porous membrane can be integrated into a microchannel without an alignment process. Using the fabrication results of the proposed methods, we describe a method for a highly accurate method of integrating a vertical porous membrane with pores capable of seeding cells into a microchannel.

**Figure 8:**
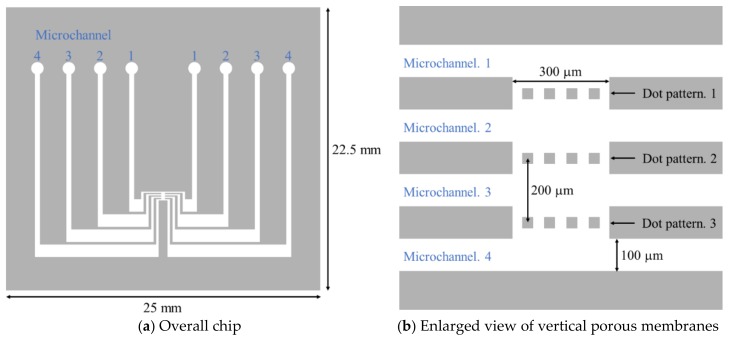
Schematics of microchannel integrated with vertical porous membranes. In the design, there are four individual microchannels. Each microchannel is connected by only the pores on the vertical porous membranes. In the vertical porous membrane, the width is 300 μm, and the interval of each vertical porous membrane is 200 μm. The dot size is 4 μm, and the interval is 10 μm for fabricating the micropores of the vertical porous membrane.

To design the dimensions of the vertical porous membranes, HepG2, a liver cell line, was adopted as the seeding cell to the membranes. Considering the HepG2 cell size, the inlet size, and microchannel height, the pore size was designed as 10 μm, and the heights of the microchannel and vertical porous membrane were over 50 μm. Using the evaluation results of the inclined oxygen ashing after inclined exposure, the dot size was set as 4 μm, and the interval as 10 μm. Figure 8 shows the design of the microchannel integrated in the vertical porous membrane. In the center of the microchannel, the dot patterns were arranged in three layers with an interval of 200 μm. Each microchannel was independent and connected only by the pores on the vertical porous membrane. By the cellular tissue covering the vertical porous membrane, multiple cell layers were arranged in parallel inside the microchannel.

The vertical porous membrane and microchannel were fabricated using inclined exposure and inclined ashing. First, a Cr layer was deposited on a glass substrate and patterned. Next, to prevent resist peel-off, a primer was spin-coated on the glass and baked at 200 °C for 1 min. SU-8 3050 was spin-coated on the substrate at 3000 rpm. After removing the solvent, inclined exposure was performed at 420 mJ/cm^2^. After developing the SU-8, inclined oxygen ashing was performed for 10 min on both sides of the vertical porous membranes. Finally, the Cr layer was etched by a wet etchant.

**Figure 9:**
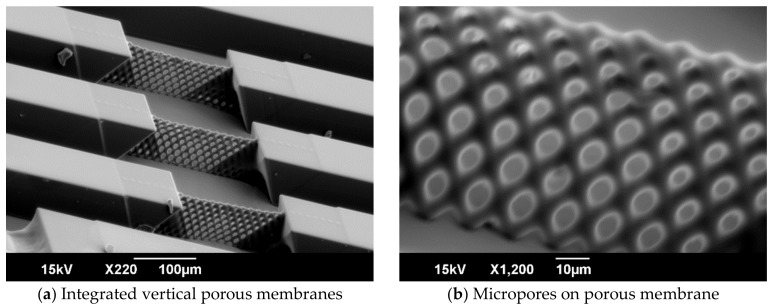
SEM images of fabricated microchannel integrated with vertical porous membranes. The microchannel was fabricated by inclined exposure with exposure dose 420 mJ/cm^2^ and double-sided inclined oxygen ashing for 10 min. The SEM images were captured at a 60° angle. The size of the micropores close to the substrate surface were 6.84 ± 0.59 μm in width and 9.40 ± 0.22 μm in height. The size of the micropores close to the top were 4.07 ± 0.30 μm in width and 4.03 ± 0.19 μm in height.

SEM images of the fabricated microchannel integrated with the vertical porous membranes are shown in Figure 9. Every micropore in the vertical porous membranes was opened, and the porous membrane did not collapse. The height of the vertical porous membrane was 79.42 μm. The maximum and minimum sizes of the micropores were 9.40 and 4.03 μm, respectively. Therefore, an integrated vertical porous membrane with height over 50 μm and pores smaller than the cell size in the microchannel was achieved.

### 4.2. Cell Culture on Vertical Porous Membranes

To evaluate the cell culture ability of the vertical porous membranes, HepG2 cells were seeded on the fabricated vertical porous membrane integrated in the microchannel. Because HepG2 cells are able to adhere to scaffold surfaces, they were used to evaluate the surface effects of the membrane on cells [24,25]. First, the fabricated microchannel was sterilized with ethanol and rinsed with phosphate-buffered saline (PBS). The microchannel was put into fibronectin (fibronectin:PBS = 10 μL:10 mL) for 30 min. After washing three times with PBS, the microchannel was covered with a sterilized PDMS chip. HepG2 cells were collected from a confluented culturing flask that had a bottom surface of 25 cm^2^. The HepG2 cells were stained with CellTracker Red (Thermo Fisher Scientific, Waltham, MA, USA). The CellTracker Red was dissolved with 12.5 μL of DMSO and mixed with 10 mL of PBS. The HepG2 cells were placed in the solution for 30 min. After washing three times with PBS and centrifugation, 5 mL of medium was added and a cell suspension was generated. A cell suspension of 200 µL was added into each microchannel by pipetting. The microchannel was set vertically inside the incubator and statically incubated for 24 h without a flow rate. The PDMS chip was removed, and the microchannel was washed with the medium. Subsequently, the microchannel was put into PBS and observed using an inverted microscope.

**Figure 10:**
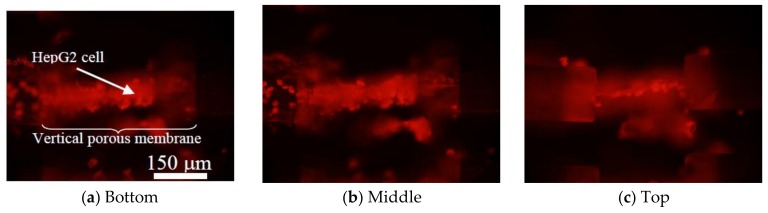
Fluorescent images of cells on a vertical porous membrane integrated microchannel. Two days after seeding, HepG2 cells adhered to the vertical porous thin film. Cell images were obtained by changing the focal length of the microscope, and the cells were observed at each height of the vertical porous membrane.

Fluorescent images of the HepG2 cells are shown in Figure 10. For observing three locations at different heights of the vertical porous membranes, three images were captured with different focal positions. The bottom is the surface of the glass substrate (Figure 10a). The middle is the center in the vertical porous membrane in the vertical direction (Figure 10b). The top is opposite to the glass substrate (Figure 10c). The HepG2 cells were observed in every focal position.

## 5. Discussion

### 5.1. Fabrication of Vertical Porous Membranes by Inclined Exposure

As a result of the fabrication by the inclined exposure of the vertical porous membrane, a structure with a dot size of 4 μm, spacing of 10 μm, and height of over 50 μm was fabricated. Typically, a vertical porous membrane is fabricated using inclined exposure [23,26,27], and a vertical porous membrane that has a pore of approximately 10 μm is usually fabricated using a pattern with a larger dot size. Owing to the large dot size and the void content, the ratio of the lattice area of the vertical porous membrane to the area of the pores produced by conventional methods is lower than that of the vertical porous membrane fabricated in this study. Regardless of the void content, the minimum pore size produced by conventional methods is generally equal to or larger than that obtained in this study. Therefore, the size of pores on SU-8 fabricated by the inclined exposure was the smallest fabricated size in this study. Additionally, this study describes the fabrication method and processing conditions of a hollow structure having a higher void content.

### 5.2. Ratio of Open Micropores Obtained by Inclined Oxygen Ashing

Using inclined oxygen ashing, a closed pore covered by a thin film of SU-8 can be opened. The thickness of the thin film sealing the pore is considered as extremely thin as compared to the lattice part. The film is extremely thin because it was produced by the reduction of the perpendicularity of exposure light and the diffusion of acid. This implies that even though the entire portion (including the lattice portion) is etched by inclined oxygen ashing, the ratio of open micropores is improved without disrupting the lattice because the thin-film portion is etched in a short time. Therefore, inclined oxygen ashing is useful for improving the porosity of a vertical porous membrane.

Suzuki et al. [28] reported an etching process for SU-8 using oxygen plasma. They used oxygen plasma to fabricate micropores using SU-8. The pores were vertical to the plasma direction. However, they did not use and verify the inclined oxygen ashing for the etching of the side structure. In this research, we report the effect of oxygen ashing for the side etching by inclined plasma irradiation.

### 5.3. Robustness of Vertical Porous Membrane Fabricated by Inclined Oxygen Ashing

A vertical porous membrane was prepared using the proposed inclined oxygen ashing such that the vertical porous membrane did not collapse during the drying process after development. The vertical porous membrane might collapse due to sticking, owing to the meniscus force during the drying process. In the drying process, meniscus force is applied to the structure, and the structure collapses or deforms. To prevent sticking, a conventional method using supercritical drying can be applied [29]. In a supercritical fluid of high temperature and high pressure, the meniscus force does not occur. Therefore, using the supercritical drying process, the influence of sticking can be reduced. Because oxygen ashing is also a dry etching method, no meniscus force is added to the structure. Therefore, the vertical porous membrane was fabricated by the proposed method without collapsing. 

Yoon et al. [23] reported that the strength of the structure is improved by exposing the vertical porous membrane from the upper surface. They found that structure collapse is prevented by reinforcing the upper part of the vertical porous membrane. However, it is necessary to perform alignment when using this method. Moreover, when a thick photoresist such as SU-8 is deposited with a thick film of several tens of micrometers or more, a difference in the height of the surface called edge beads occurs. The edge beads generate an undesirable contact gap. The contact gap is the distance between the photosensitive material surface and the mask pattern surface, which lowers the fabrication accuracy. Therefore, it is difficult to construct a high-precision alignment from the top surface direction as well as a high-resolution structure. Because the proposed method is assembly-free, it has the advantages that not only can fine structures be fabricated by a short-term process, but also fabrication errors do not occur.

Compared to the mesh structure fabricated in Yoon’s research, the significant difference here is the area ratio of the pore/lattice. The pore size in Yoon’s research and our research was the same. However, in Yoon’s research, they fabricated lattices larger than 10 μm to prevent mesh collapse by sticking. Sticking occurs during drying. Because our process does not involve drying before the mesh becomes finer by oxygen plasma, a vertical porous membrane with a high pore/lattice ratio can be fabricated.

### 5.4. Cell Culture on Vertical Porous Membrane

The seeded HepG2 cells on the SU-8 surface adhered to the surface of the vertical porous membrane in this study. Cell culture on SU-8 has been performed previously, and adherent cells adhered to the structure, similar to our results [16,17,18]. Meanwhile, other studies have improved the cell adhesion rate using oxygen ashing on SU-8 [30]. The primary reason for applying inclined oxygen ashing is to improve the porosity. However, inclined oxygen ashing is considered applicable for treating the surface of SU-8 for cell culture.

The seeded HepG2 cells were observed from the bottom to the top of the vertical porous membrane. During the cell culture in the microchannel, the PDMS-covered chip stood upright in a culture dish. Therefore, the vertical porous membrane was located on the bottom of the microchannel, and the seeded cells were maintained in contact with the membrane. Because they were washed with PBS before the observation, cells that did not adhere to the vertical porous membrane were removed. Therefore, the seeded cells adhered to the surface of the vertical porous membrane with sufficient adhesive strength. By culturing for a longer period of time, the boundary is expected to be constructed by the cell layer, and metabolites can be acquired separately between each microchannel that is separated by the mimicked barrier tissues.

## 6. Conclusions

In this paper, we proposed an integration method using inclined exposure and inclined oxygen ashing for fabricating microchannels with vertical porous membranes made of the thick photoresist SU-8. Inclined oxygen ashing was demonstrated to be useful for improving the ratio of open micropores of the vertical porous membrane. Using the proposed methods, a vertical porous membrane having a pore size smaller than the cell size and a membrane height of 50 μm could be fabricated. Moreover, the vertical porous membrane was integrated into a microchannel through an assembly-free process. When HepG2 cells were seeded on the prepared vertical porous membrane, adhesion of the HepG2 cells to the surface of the membrane was observed. Therefore, the proposed integration method and the fabricated vertical porous membrane are expected to be applicable to biomicrodevices.
